# Post-Graduate Dental Association

**Published:** 1892-07

**Authors:** 


### PROCEEDINGS.

### Post-Graduate Dental Association.

The third annual meeting of the Post-Graduate Dental Association of the United States was held in Chicago, Friday and Saturday, April 29th and 30th.

The first day was devoted to clinics which were given at the Chicago College of Dental Surgery. These proved to be a very interesting part of the programme, and consisted of:

Experiments with anæsthetics upon the lower animals. Dr. W. C. Barrett, of Buffalo, N. Y. Dr. Barrett held the attention of a large number of the members for several hours. His demonstrations of the actions of nitrous oxide were especially interesting.

Dr. James A. Swasey, of Chicago, inserted a gold inlay filling in an incredibly short space of time. In the words of the Doctor, "It is worth something to know how to do this in order that we may save time and pain and discomfort for our patients."

One of the features of the day was the exhibition of regulating appliances by Dr. Angle, of Minneapolis, Minn. All his well known appliances, together with many new ones, were shown with explanations and demonstrations regarding their construction. It is surprising how simple and easy it is to make many of these which at first thought seem complicated and difficult. The Doctor is indeed master of his specialty.

Dr. H. C. West, of Chicago, demonstrated his method of root filling.

Dr. J. A. Dunn, of Chicago, demonstrated his treatment of abscesses with fistulous opening, exhibiting special appliances.

In the evening a rousing meeting was held, to which a general invitation was extended to the dentists of Chicago. The speaker of the evening was Dr. W. C. Barrett, who entertained the meeting in his usual brilliant manner. He dwelt at some length upon the subject of home study, congratulating the Post-Graduate Dental Association upon the work it has been doing. The meeting was also addressed by Drs. Brophy, Ottofy and Clifford, of Chicago, Drs. Angle and Martindale, of Minneapolis, Minn., and Dr. Owen, of Broadhead, Wis. In behalf of the management, Dr. Ottofy outlined in brief the course of home study as proposed, and which will be open early in the summer.

SECOND DAY.

The meeting was called to order at the Leland Hotel at 10 A. M.

The President presented his annual address, recapitulating the work that had been accomplished this year. While to those who had not been in intimate contact with the work little advancement might seem to have been made, yet it was shown that much important work had been done, that they were a long way in advance of last year, with a system of home study almost ready to put into operation, and that an earnest and widespread interest had been developed among many of the best dentists of the country as well as all along the rank and file generally.

Dr. Tuller made several suggestions for the future, which were discussed, among them a suggestion as to what part they would take in the Columbian Dental Congress next year, recognizing that owing to their youth and incomplete plans of work, they could not take any important part. The appointment of a committee to investigate and suggest plans, and ways and means was left to the President. The presentation of models, appliances and incidents of office practice gave rise to animated discussion, which brought out many new and valuable ideas.

Dr. Nicholson, of Wisconsin, presented a model of a very interesting and difficult case of irregularity which he had successfully handled.

Dr. Julian, of Indiana, presented a beautiful and difficult piece of crown and bridge-work.

Dr. Owen, of Wisconsin, illustrated a method he had used to correct a faulty occlusion, which was highly appreciated.

Dr. Tenney gave demonstrations of the most advantageous positions to occupy in performing difficult operations.

Dr. Ottofy followed in the same strain, demonstrating the many advantages of a position at the left of the chair. But it was observed that Dr. Ottofy had the special advantage in such cases in that he could use his left hand with almost equal facility with the right.

Dr. Tuller claimed that this could be attained by almost any-one, particularly the young student, if dilligent efforts were made, and thought our colleges and teachers should impress and even enforce it upon students, insisting that in many operations the handling of instruments should be done with the left as well as the right hand, making them thereby better manipulators and more skillful dentists.

Dr. Fahr, of Wisconsin, presented diagrams and models showing a method he had adopted in restoring abraded molars, employing screw anchorages and building up with platinum and gold.

The meeting this year was called two months earlier than last to correspond with the change made by the Chicago College of Dental Surgery in holding its practitioners' course, and as the charter, as adopted at the last meeting, does not permit the election of officers until the end of the year, this order of business was suspended and the meeting adjourned, subject to the call of the President and Secretary. A meeting will probably be called during the latter part of June.

